# Lessons Learned about Human Stem Cell Responses to Ionizing Radiation Exposures: A Long Road Still Ahead of Us

**DOI:** 10.3390/ijms140815695

**Published:** 2013-07-29

**Authors:** Mykyta Sokolov, Ronald Neumann

**Affiliations:** Nuclear Medicine Division, Department of Radiology and Imaging Sciences, Clinical Center, National Institutes of Health, 9000 Rockville Pike, Bethesda, MD 20892, USA; E-Mail: rneumann@mail.nih.gov

**Keywords:** human embryonic stem cells, human adult stem cells, ionizing radiation, radiation toxicity

## Abstract

Human stem cells (hSC) possess several distinct characteristics that set them apart from other cell types. First, hSC are self-renewing, capable of undergoing both asymmetric and symmetric cell divisions. Second, these cells can be coaxed to differentiate into various specialized cell types and, as such, hold great promise for regenerative medicine. Recent progresses in hSC biology fostered the characterization of the responses of hSC to genotoxic stresses, including ionizing radiation (IR). Here, we examine how different types of hSC respond to IR, with a special emphasis on their radiosensitivity, cell cycle, signaling networks, DNA damage response (DDR) and DNA repair. We show that human embryonic stem cells (hESCs) possess unique characteristics in how they react to IR that clearly distinguish these cells from all adult hSC studied thus far. On the other hand, a manifestation of radiation injuries/toxicity in human bodies may depend to a large extent on hSC populating corresponding tissues, such as human mesenchymal stem cells (hMSC), human hematopoietic stem cells (hHSC), neural hSC, intestine hSC, *etc.* We discuss here that hSC responses to IR differ notably across many types of hSC which may represent the distinct roles these cells play in development, regeneration and/or maintenance of homeostasis.

## 1. Introduction

Scientific research into human stem cells (hSCs) has gained enormous momentum recently, in part because of the promise that these cells hold in the field of cell regenerative and replacement therapies. Although thousands of scientific studies have been published illuminating different aspects of hSC biology, the responses of hSC to genotoxic stresses in general, and to ionizing radiation (IR) in particular, are still far from being understood in full detail. Part of the problem lies in a lack of some very basic knowledge regarding hSC nature, and their identity; other issues involve the natural scarcity of hSC within the body, and problems of their isolation for studies *in vitro*, difficulties of establishing the continuous cultures of hESC, etc, and as such can be regarded as being methodological; there are ethical and other issues at play too. For example, moral biases towards possible human embryo destruction for the purpose of generating human embryonic stem cells (hESCs) were intensely discussed and raised a general concern among the broad community. Human ESCs are derived from the inner cell mass of the preimplantation embryos. The techniques for their isolation and maintenance, and initial characterizations of hESC were first reported in 1998 by Thomson *et al.* [1]. HESCs were shown to maintain the pluripotency in culture under non-differentiating conditions [2,3]. Such cells demonstrate a stable developmental potential by forming committed cell lineages representative of all three embryonic germ layers, including adult hSCs.

It is thought that the majority, if not all, organs and tissues of an adult human contain hSC/progenitors at the apex of the hierarchical organization; and these adult hSCs are generally considered to be multipotent. Human mesenchymal stem cells (hMSCs) were first discovered in 1968 [4]. hMSCs represent an adherent fibroblast-like population in the human bone marrow capable of differentiating into bone, cartilage, adipose, *etc.* The populations of hMSCs with similar characteristics have been isolated from other tissues, such as adipose tissue, peripheral blood, umbilical cord, amniotic fluid, adult brain [5], *etc.* Therefore, these cells are thought to populate various stromal compartments of the human body, and hence sometimes are known as a mesenchymal stromal cells, or multipotent progenitors. Research into hMSCs biology has been hampered in part because of a lack of unique definitive hMSC surface markers. To overcome this limitation, the International Society of Cellular Therapy defined hMSCs based on three following criteria: firstly, hMSCs must be able to adhere to plastic surface under standard tissue culture conditions; secondly, hMSCs must express certain markers, including CD73, CD90, and CD105, and lack the expression of other markers, such as CD45, CD34, CD14, CD79alpha or CD19 and HLA-DR surface molecules; and, finally, hMSCs must be capable of differentiating into osteoblasts, chondroblasts, and adipocytes under appropriate *in vitro* conditions [6]. In addition, hMSCs are relatively easy to obtain and are proliferative under defined culture conditions, and hMSCs are not potent elicitors of immunoreactivity in the host upon both local transplantation and/or systemic administration. Further complication into the field was brought by studies showing that hMSC possess characteristics of pericytes, such as expression of CD146 [7,8], even though more recent research does not seem to fully support this claim [9]. Regardless these controversies, it is established that bone-marrow residing hMSC support the regulation of human hematopoietic stem cells (hHSCs) by physical interaction with them [10].

It is known that hematopoietic system homeostasis in humans is kept in order by the fine interplay between proliferation, differentiation, and death of a quite small number of long-term surviving, self-renewing stem cells, which give rise to the fully mature blood cells. Human hematopoietic stem cells (hHSCs) were first reported to be isolated in 1995 [11]. The vast majority of the hHSCs is in the bone marrow; it is the bone marrow microenvironment that is chiefly responsible for proliferation, differentiation and migration of these cells. These hHSCs were shown to be capable of secondary colony formation, and produced both lymphoid and myeloid progeny. More recently, CD49f was shown to be a hHSC defining marker; it is CD49f (+) single hHSCs that appear to be capable of replenishing mature human blood cells through downstream lineage-restricted intermediates [12]. On the other hand, a single marker may not fully define the hHSC compartment; indeed, CD49f could also mark human colon cancer stem cells [13], and hence may not be unique to hHSCs.

Such a linear hierarchical model may not exist in other human tissues. For example, the intestinal tract is known to consist of two anatomically and functionally distinct organs, such as the small intestine and the colon [14]. The architecture of the crypt-villus unit is repetitive and the crypts possess the intensive self-renewal characteristics which may make the intestinal tract a good model to study hSC biology [15]. However, the existence of distinct non-overlapping subpopulations of intestinal stem cells in humans (hISCs) postulated earlier complicates the research based on marker lineage tracing. Therefore, some novel approaches may need to be implemented in order to gain novel insights into the complexity of hISCs, and their responses to genotoxic stresses. Non-linear hierarchical architecture seems to be inherent to other complex tissues, such as human brain [16].

One of the key deterministic consequences of radiation injury/toxicity is the development of IR-induced clinical syndromes, the nature of which depends highly on IR dose. It is widely known that relatively modest doses of IR exposures in humans elicit hematopoietic syndrome, higher doses trigger the development of gastrointestinal syndrome, and even higher doses lead to central nervous system paralysis. In survivors, lower doses of IR exposures later may result in radiogenic cancers virtually in all locations in the body, but these are probabilistic in nature. Therefore, given the paramount importance for human health and IR risk prevention strategies, the clarification of whether the hSCs residing in organs/tissues are responsible for such outcomes becomes a major issue for the scientists to address.

## 2. Human Stem Cell Radiosensitivity

It is well established that the radioresponses of differentiated somatic human cells depend to a large extent on both the physical characteristics of IR exposures, such as dose of IR, dose-rate, linear energy transfer (LET), and the biological characteristics of the exposed tissue (microenvironment, proliferative state of cells, *etc.*). Many outcomes of such IR exposures were examined thus far in different types of somatic cells, including apoptosis, necrosis, autophagy, senescence, cell cycle arrest, and many others. In marked contrast, the radioresponse of hSCs was examined in a relatively few studies, warranting further research into the mechanisms underlying the survival/death decisions in irradiated human pluripotent and multipotent cells.

### 2.1. Radiosensitivity of Human Embryonic Stem Cells

**Figure 1:**
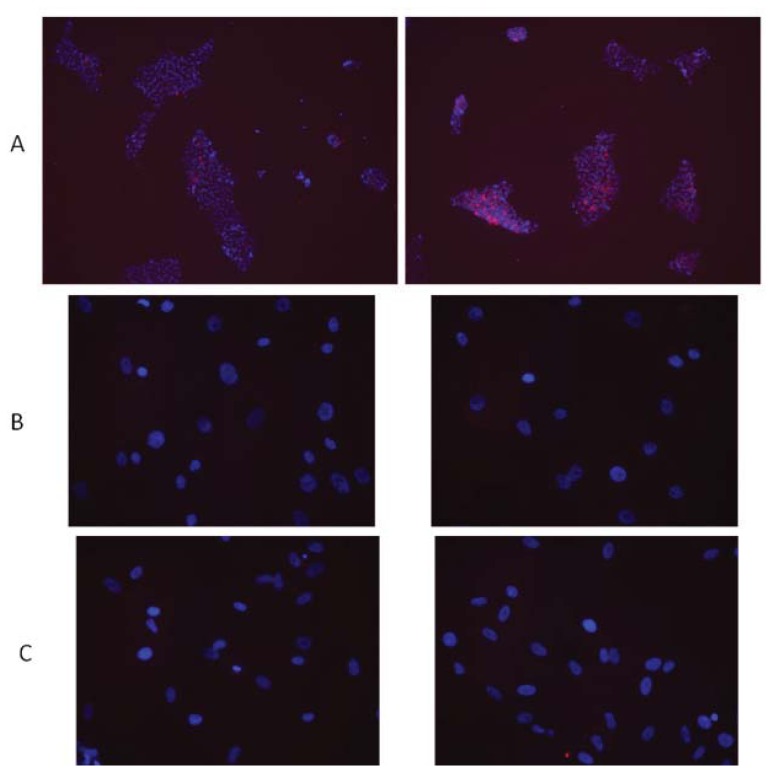

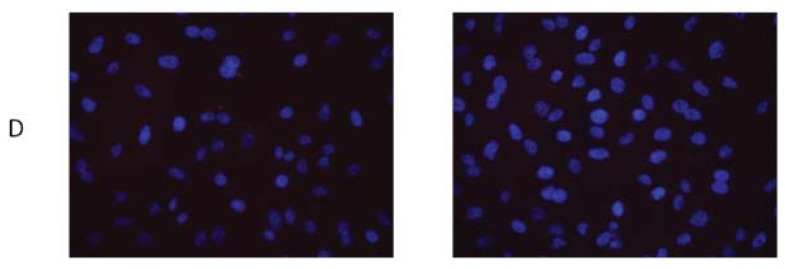
Radiation toxicity for various types of human cells assessed by induction of apoptosis. Shown below are the immunocytochemistry data for cell cultures 6 h post IR exposures (red—cleaved caspase 3, blue—nuclei, DAPI staining); left column—mock irradiation, right column—1 Gy. (**A**) H9 hESCs; (**B**) hMSCs; (**C**) BJ normal foreskin fibroblasts; (**D**) HeLa tumor cells.

Human embryonic stem cells were observed to undergo apoptosis at doses of IR exceeding the low-dose range; and the phenomenon is dose-dependent [17]. The activation of cleaved caspase 3 in hESC cultures is readily observed as early as 6 h following exposures in 1 Gy (Figure 1A).

Programmed cell death manifests itself in the formation of holes in hESC colonies and a dramatic increase in the number of detached cells [18,19]. Quantitative estimates suggest that as many as two-thirds of H1 hESC are dying at 7 h post 5 Gy of gamma-radiation [20]. Interestingly, the surviving fraction of irradiated hESCs preserves the key characteristics of pluripotent cells, such as an ability to self-renew and give rise to derivatives of all three germ layers [18,19,21].

Programmed cell death in hESC cultures appears to be the primary outcome of not only exposures to IR, but also to other types of genotoxic stressors such as UV [22]. Not surprisingly, p53 was found to be rapidly activated by UV; and it governs apoptosis by activating the cell death mitochondrial pathway through caspase 9 [22].

The mechanisms of apoptosis in hESCs recently became a focus of intense research; partly because of the promise these pluripotent cells hold for regenerative medicine and the practical need to understand why these cells are so sensitive to external cues. It was found that the large faction of hESCs undergo the programmed cell death in response to thawing following cryopreservation [23], as a result of heat shock response [24], and upon exposure to genotoxic substances, such as etoposide [25]. The high efficacy of apoptosis induction in hESCs has been shown to depend on multiple mechanisms, including the expression of elevated levels of multiple pro-apoptotic proteins [26]; and constitutively active Bax sequestered at Golgi rapidly translocating to mitochondria to trigger the cell death program [27]. The unique abbreviated cell cycle of hESCs [28–30], and the absence of functional G1 and S phase checkpoints operating in these pluripotent stem cells following IR exposures and replicative stress conditions [31] are believed to be at least in part also contributing to the hypersensitivity of hESCs cultures to apoptosis induction. Intriguingly, the induction of apoptosis in irradiated hESC cultures was recently shown to be cell cycle-dependent, occurring preferentially in S phase in these cells [27]. The elimination of hESC with radiation damage by means of apoptosis in S phase is important for several reasons; first, it is believed to be the longest cell cycle phase in hESCs which may account for up to half of all cell cycle timing, and second, it may serve to remove the compromised hESCs with damage inflicted during G1 phase when error-free homologous recombination DNA repair is non-functioning. These differences in unique details underpinning molecular mechanisms of apoptosis operating in hESCs and other types of human cells may explain the propensity of hESCs to undergo programmed cell death in response to genotoxic stresses including IR exposures.

Many biological pathways and cellular components were shown to be involved in induction/execution of apoptosis, on one hand [32,33], and protection of hESCs from the programmed cell death, on the other hand [34–40]. However, there is still no coherent picture on all the details of radiation-induced apoptosis in hESCs; it is plausible that IR exposures shift the delicate balance between pro-survival and pro-death choices in stressed hESC cultures in favor of the latter, even though the marked heterogeneity of hESC cultures observed earlier may explain why some subpopulations of hESC survive after genotoxic stress whereas others within the same cultures die [18,21].

### 2.2. Radiosensitivity of Human Mesenchymal Stem Cells

It is known from clinical practice that hMSCs from patients, exposed to whole body irradiation followed by allogenic bone marrow transplantation, presented a complete host profile implying that the hMSCs in their niches could be radio-resistant [41]. The data on radiosensitivity of hMSC are still limited; just a few published reports provide some information regarding the survival/cell fate of irradiated hMSC derived from different body locations [42–46]. In general, hMSCs were found to be relatively resistant to IR exposures [42,47,48]. Importantly, hMSC ectopically expressing hTERT were less radiosensitive than regular telomerase-negative hMSCs [49]. Intriguingly, the effect of hypoxia on hMSCs survival after IR is limited [48]; it could be unexpected, since hypoxic conditions usually confer radioprotective properties on irradiated cultures. Part of the explanation could be that the hMSC niche within the body is believed to be quite hypoxic, so hMSCs may be already equipped with hypoxia-related protecting signaling machinery to deal with IR insults even under normoxic conditions.

The dose required to reduce the fraction of surviving cells to 37% for hMSCs was shown to be about 2 Gy [42]. We found a lack of robust apoptosis induction in hMSC exposed to doses of IR up to 10 Gy (Figure 1B) and [50]. More recently, it was confirmed that IR exposures with high doses significantly reduces hMSC proliferation [51,52], and DNA synthesis [52] but has no substantial effect on cell viability (more than 80% even after 20 Gy of IR) [51]. The inhibition of proliferation of hMSC was also observed after 1 Gy of both low-LET X-rays and high–LET ^56^Fe ions [44].

Caspases 3/7, 8 and 9 were not induced by 6 Gy and 20 Gy of IR [51]. In culture, spindle-shaped hMSCs display a lag phase of growth, followed by a log phase, and finally a plateau state. The average number of population doublings for bone marrow-derived hMSCs was found to be 38 ± 4, at which time the cells finally become senescent [53]. Replicative senescence of hMSCs could be regulated by multiple pathways, including cyclin-dependent kinases inhibitor 2A (p16(INK4a)) pathway [54], Notch signaling [55], induction of specific miRNAs [56], among others; however, the mechanisms of IR-induced senescence of hMSCs have not been thoroughly studied. Some recent data suggest that IR-induced premature senescence is associated with the increase in p16 protein and increased activity of senescence-associated β-galactosidase [51]; the propensity of hMSC to undergo IR-induced senescence was at least partly associated with low intrinsic antioxidant activity in hMSCs (at least 3-fold lower compared with fibroblasts and cancer cells) [52]. Extensive proteomic and metabolomic profiling of hMSC cultures undergoing oxidative stress induced-senescence by high doses of hydrogen peroxide showed decreases in glycine and proline and increases in choline, leucine, NAD+, and UDP-glucose; and the levels of ANXA2 and PSMA1 were found to be significantly modulated [57].

Several cellular mechanisms were implicated in relative hMSCs radioresistance, including DNA damage response (DDR), like ATM protein phosphorylation, cell-cycle checkpoint activation, double-strand break (DNA DSB) repair, and the antioxidant capacity for reactive oxygen species (ROS) detoxification [42]. It was shown that hMSCs possess a quite robust antioxidant system and active DNA repair that may promote their radioresistance. Human MSC cultures are equipped with the capabilities of differentiating into osteocytes, adipocytes, and hepatocytes following IR exposures. Relatively modest doses of IR (up to 1 Gy of both low-LET X-rays and high–LET ^56^Fe ions) fail to impair the osteogenic differentiation process of hMSCs [44]; however, high doses of IR (4–12 Gy) reduce osteogenic and adipogenic activities in hMSC cultures up to 50% [43]. Importantly, hMSCs predifferentiated into adipocytes showed sensitivity to IR exposures [48]. In general, even though recent work has yielded some mechanistic insights into the radiosensitivity and cell fate of irradiated hMSC, many unknowns still remain. For example, the involvement of BMP-2, SIRT1 and DNMT1 in radioresponses of hMSCs is not clear; previously, these proteins were shown to be critical for hMSC proliferation, self-renewal and/or maintenance of multipotency [58–60]. There is still some controversy in the literature regarding the differentiation capabilities of stressed hMSCs; some evidence suggests that with prolonged time in culture hMSCs accumulate DNA damage resulting in the loss of multipotency in these cells [61], which is in marked contrast to preservation of multilineage differentiation in hMSCs after IR exposures likewise inducing DNA damage. It could be that the type/extent of DNA damage dictates whether or not stressed hMSCs in culture still retain the ability to undergo differentiation; further studies may help to clarify this apparent contradiction.

### 2.3. Radiosensitivity of Human Hematopoietic Stem Cells

Human hematopoietic stem cells (hHSCs) were found to be exquisitely sensitive to radiation, responding with massive apoptosis to even modest doses of IR exposures (about 50% of cell killing following 1 Gy) [62]; expression of vascular endothelial growth factor (VEGF) somewhat protected hHSCs from dying [62]. In cell cultures, it was shown to be essential to add hematopoietic cytokines to growth media to support survival of hHSCs [63]. Importantly, high-LET carbon ion IR resulted in an enhanced apoptosis and chromosomal aberration yield in hHSCs compared to low-LET X-rays, even though RBE for carbon IR was only 1.4–1.7 suggestive of high radiosensitivity of hHSCs [63]. The radiation toxicity for hHSCs was found to be comparable to those found in peripheral blood lymphocytes; apoptotic induction is both dose- and time-dependent [63]. Proteins of Bcl-2 family are responsible for the maintenance of the fine equilibrium between apoptosis and cell survival capabilities in hHSCs [64]; it can be hypothesized that IR exposures of hHSCs shift the balance in favor of pro-apoptotic Bcl-2 members. The apoptotic induction in hHSCs was shown to depend not only on Bcl-2 but also on p53, and ASPP1 [65]. The hHSCs are in general kept in quiescent state by low levels of TGF-beta, which in turn (i) promotes expression of one of the key markers of hHSCs, namely mucin-like protein CD34 [66,67]; (ii) induces CDKN1A mRNA; and (iii) downregulates cytokine receptors [68]. The expression of cyclin C (*CCNC*) was recently shown to serve as a regulator to promote transition of G0 phase hHSCs to G1 phase [69]. Therefore, the quiescence of hHSCs seems to be reinforced by multiple mechanisms. However, the scarcity of hHSCs within the bone marrow and the apparent heterogeneity of hHSC populations possessing distinct molecular characteristics (up to five subsets) based partly on expression of CD114 [70] complicates research studies and the interpretation of results obtained.

### 2.4. Radiosensitivity of Human Neural Stem Cells

Human neural stem cells were previously shown to be responsive to both modest and high doses of IR (1–5 Gy) [71]. Under regular cell culture conditions, hNSCs are characterized by doubling times of approximately 28 h, preserving the undifferentiated state up until 10 passages. Human NSCs were found to be radiosensitive, since IR exposures with a relatively modest dose of 1 Gy reduce cell numbers by three- to fourfold; and the effect of cell killing was dose-dependent within a 1–5 Gy dose range [71]. Doses of 5 Gy resulted in only 20% survival of hNSC derived from iPSCs [72]. Apoptosis was clearly implicated in hNSC killing after IR; the level of programmed cell death increased fourfold over sham-treatment at 12 h post 5 Gy of IR exposures [71]. At the same time, the doses of IR up to 2 Gy fail to induce spontaneous differentiation in surviving multipotent hNSC. Importantly, hNSCs were found to preserve the cognitive abilities of IR-exposed brains of mammals upon engraftment [73]; it is noteworthy that there is a preference for hNSCs to undergo differentiation towards astrocytic lineages (up to 46%) in a long-term engraftment studies with 12% of hNSC surviving four month after transplantation [73,74]. Human NSCs that survived IR exposures were demonstrated to be prone to undergo cellular senescence acquiring astrocytic properties [75]. Interestingly, in another study the mammalian astrocytes were shown to be radioresistant lacking the ability to mount functional DNA damage response (DDR) signaling still being DNA repair proficient [76]. Surprisingly, the metabolic activity of surviving multipotent hNSC was shown to increase with doses up to 5 Gy; this coincides with the modest dose-dependent increase in oxidative stress (1.2–1.3-fold over control) observed in these hNSC 12 h post IR [71].

## 3. Effects of Ionizing Radiation Exposures on the Cell Cycle of Human Stem Cells

The cell cycle perturbations of IR-exposed normal differentiated and cancer human cells have been extensively studied. However, the molecular details of the cell cycle in pluripotent and multipotent hSCs have only recently begun to emerge [17].

### 3.1. Cell Cycle Alterations in Irradiated Human Embryonic Stem Cells

It was shown that hESCs cultures are inherently characterized with a relatively short cell cycle (15–16 h) compared with human differentiated somatic cells [28]. In a marked contrast with the latter, the duration of the hESC cell cycle in G1 phase is substantially abbreviated, lasting only about 2.5–3 h in unperturbed cell cultures [28].

Human ESCs and fully differentiated cells share many cell cycle markers in common; however, the molecular details of the cell cycle governing network were demonstrated to be different. For example, the expression of G1 phase-related *CCND2* and *CDK4* genes was found to be elevated in hESCs [28] and many of RB and E2F family genes consistently show unique patterns of expression in hESCs [77]. Interestingly, the distinct sets of E2F and RB family transcription factors are predominantly expressed either in unstressed (RB2, E2F4, E2F5) or IR-exposed hESCs (RB1, E2F5, E2F6) [77]. Another protein, heterogeneous nuclear ribonucleoprotein A2/B1 (hnRNP A2/B1) was enriched in hESCs, and regulates the G1/S transition of the hESC cell cycle through the repression of p27 and control of p53 and Chk1 activity [78]. An additional layer of complexity arises with the recent discoveries showing that the hESC-specific miRNA signaling network is capable of regulating the progression of hESCs through the cell cycle and maintenance of self-renewal: the levels of WEE1 kinase, which is a target of *miR-195*, controls the rate of hESC division, whereas the expression of *CDKN1A* is kept at low levels by *miR-372* for hESC division to proceed [79]. More recently, additional miRNAs, such as *miR-302* family genes, were implicated in direct regulation of the levels of p21 protein in hESCs, thus affecting the cell cycle machinery through the G1/S checkpoint that is often described as being non-operational in hESC [19]. One of the most important signaling networks underlying histone expression and chromatin assembly to promote cell renewal, namely the HiNF-P/p220 gene regulatory pathway, was shown to be operative in hESCs [80]. Additional studies examining the mechanistic details of abbreviated cell cycle in hESCs revealed that the G1 phase in hESCs is shortened in large part by contraction of late G1 [81].

Accelerated upregulation of histone genes which is a prerequisite for DNA replication is one of the key gene expression programs in late G1 in hESCs, and it was found to be determined by a hESC-specific chromatin structure with atypical distribution of epigenetic histone marks on a chromatin fibre [82]. In irradiated hESCs cultures, histone gene expression downregulation was observed, indicating coordinated changes in the cell cycle-gene expression machineries operative in pluripotent human stem cells [77].

In summary, hESCs are equipped with unique G1 cell cycle profile utilizing distinct cell cycle machinery components that bypasses “conventional” E2F/pRB-dependent growth control to maintain pluripotency and progress through the cell cycle as compared with other types of human cells including fully differentiated human somatic cells [29,30,83,84].

### 3.2. Changes in Cell Cycle in Human Mesenchymal Stem Cells Exposed to IR

The doubling time of unstressed hMSCs is found to be approximately 38 h [47]. The non-irradiated hMSCs had 70%–71% of their total cell population in G1 phase, 15%–21% in S phase and 9%–14% in G2 phase of the cell cycle [42,47]. Low-dose IR (0.1 Gy) did not result in noticeable changes in hMSC cell cycle distribution compared to sham-treated cell cultures, whereas higher 1 Gy exposure induced significant cell cycle alterations [44]. Both low-LET X-rays and high-LET ^56^Fe ions caused a decrease in the population of S phase cells after 1 Gy. 1 Gy of ^56^Fe ion IR exposures also caused a significant increase in G2/M phase cells. Moreover, 1 Gy ^56^Fe ions decreased the fraction of cells in G1/G0 phase, whereas 1 Gy X-rays showed the opposite trend. In general, both X-rays and ^56^Fe ions seriously hindered the entry into S phase after 1 Gy dose, but high-LET ^56^Fe ions were a more potent inhibitor of mitosis compared with X-rays [44].

In another sets of experiments, at 36 h post 9 Gy of IR exposures hMSCs had 75% G1, 3% S, and 25% G2/M phase distribution, respectively [42]. These data implicate that both G1/S and G2/M cell cycle checkpoints operate in hMSCs following IR; however, there is no replication arrest in IR-exposed hMSCs [47].

Recently, a more detailed analysis of cell cycle of irradiated bone marrow (BM)-hMSCs showed a substantial accumulation of cells in G2/M cell-cycle phase (36% and 35%; control 12%) after 6 Gy and 20 Gy, which was partially relieved on the sixth day post exposure (6 Gy) [51]. The data indicate that hMSCs derived from both bone marrow and periodontal ligaments preferentially accumulate in the G2 phase of the cell cycle in response to high doses of IR. The percentage of cells arrested in G2 phase and also the duration of the cell cycle arrest is found to be clearly dose-dependent.

IR is known to induce upregulation of cyclin-dependent kinase inhibitor, p21 protein, in a variety of differentiated human somatic cells. The increase in p21 protein expression is observed at first day after IR and lasts for more than six days after 20 Gy of IR exposures in hMSCs; interestingly, the level of *CDKN1A* mRNA remains elevated up until 13 days post 20 Gy [51]. The decrease in the amount of p21 in IR-exposed hMSCs coincides with an increase in another inhibitor of cyclin-dependent kinases, protein p16. This is found to occur starting on day 6, with the maximal upregulation of p16 observable after 13 days post 20 Gy. In marked contrast to *CDKN1A*, no increase in *CDKN2A* mRNA occurs. The upregulation of p16 is accompanied by a decrease in the phosphorylation of retinoblastoma protein (RB) at serine 780, which is detected after 13 days in irradiated hMSCs [51]. Importantly, RB1 is indispensable for normal cell cycle progression in hMSCs [85], and RB1 downregulation/silencing leads to senescence. Therefore, the cell cycle-relevant molecular changes observable after high doses of IR in hMSCs result in the onset of stress-induced senescence program, which is probably dose-dependent too.

### 3.3. Changes in Cell Cycle in Human Hematopoietic Stem Cells Exposed to IR

The cell cycle checkpoints were apparently not activated in hHSC cultures *in vitro* even after 4 Gy of IR exposures [63]; this was suggested be a unique characteristic of these cells. However, only about 50% of hHSC were stimulated to enter the cell cycle from the state of quiescence regardless of IR exposures, hence the data need to be interpreted with caution.

### 3.4. Changes in Cell Cycle in Human Neural Stem Cells Exposed to IR

The data on cell cycle alterations in irradiated hNSC are scarce. The cell cycle distribution of hNSC was shown to be about 65% G1 phase, 25% S phase, and 10% G2/M phase [71]. Following 5 Gy of IR exposures, the changes in G2 phase of cell cycle were dramatic; at 24 h post exposures, almost 30% of hNSC were in G2/M (threefold over control). 60% of irradiated hNSC were in G1, and somewhat less than 10% resided in S phase [71]. These results may indicate that hNSC engage mostly G2/M checkpoint after high doses of IR.

## 4. Effects of Ionizing Radiation Exposures on DNA Damage Response of Human Stem Cells

The DNA damage response (DDR) constitutes a key component of cellular alterations governing changes in DNA repair, activation of cell cycle checkpoints and cell metabolism after IR exposures. However, only recently have the molecular details of DDR begun to be described in hSC compartment.

### 4.1. DNA Damage Response in Irradiated Human Embryonic Stem Cells

One of the earliest molecular events required for inducing DDR in response to DNA double-strand breaks (DSBs) is the activation of the ataxia telangiectasia mutated (ATM) signaling pathway. ATM kinase is phosphorylated and localized to the sites of DNA DSBs within 15 min of IR exposures in hESC cultures [19]. The timescale of activation of ATM measured by phosphorylation of kinase at serine 1981 was described as follows: ATM activation plateaus until four hours after IR exposures, and then the ATM levels decline, but still remain elevated over those in mock-treated hESC cultures for at least 24 h [19]. ATM induction led to activation of its substrates, such as p53, Chk2, and Nbs1 by means of phosphorylation. For example, phosphorylation of Chk2 at threonine 68 reached its maximum level at one hour after IR and then gradually diminished; Nbs1 phosphorylation at serine 343 followed a similar pattern [19]. Importantly, the relative role of ATM in H2AX phosphorylation in IR-exposed hESCs is not firmly established; some studies suggest the role for ATR in this process [86]. The number of γ-H2AX ionizing radiation-induced foci (IRIF) increased dramatically within minutes following IR and returned almost to control levels in unexposed hESCs cultures within 24 h, which is close to what have been observed in fully differentiated human cells too. The same temporal pattern was reported for 53bp1 IRIF too [87].

One of the most important components of DDR integral to both cell cycle arrest, and cell fate decision following genotoxic stresses is the p53 protein [88,89]. P53 exerts its effects through multiple posttranslational modifications, including phosphorylation on serine 15 and 20. These activation marks on p53 were readily observed within the first hour after IR exposure of hESCs, then peaked by two hours following IR, and remained elevated above the levels seen in unstressed hESC cultures for 24 h [19].

Such an activation of key DDR proteins after IR in hESCs resulted in a temporary cell cycle arrest at the G(2)/M phase after 2 Gy dose of gamma-radiation [19]. ATM is essential in establishing G(2)/M arrest since ATM knockdown led to a decrease in a number of arrested cells just 2 h post-IR exposures [19]. It is worth noting that hESCs resume the cell cycle approximately 16 h after IR exposures, bearing a 4-fold higher yield of aberrant mitotic figures compared with sham-exposed hESCs cultures. Interestingly, the mitotic spindle checkpoint may function in hESCs, but could be uncoupled from apoptosis [90]. It is only at 48 h post-IR that the cell cycle distribution resumes a pattern close to that observed in non-irradiated cells.

We, and others, reported that hESCs lack G(1)/S phase arrest after IR exposures [19,87]. Unexpectedly, other types of genotoxic stresses, such as UVC exposures of hESCs, were shown to result in G(1)/S phase arrest before DNA synthesis; this effect was correlated with a decrease in CDK2 activity [91]. However, p21, the key component of G(1)/S checkpoint induction in fully differentiated human cells, was found to be not implicated in UVC-exposed hESCs. Therefore, DDR appears to be highly context-dependent in hESCs.

It was shown that the levels of mRNA for p21 were strongly (about 15-fold) increased by 5 Gy of IR exposures in hESCs cultures [20]; but the amount of p21 protein in hESCs has remained almost flat. Therefore, the *CDKN1A* gene is robustly expressed at the level of transcriptional regulation post-IR exposures but the translation of p21 is not robust enough in IR-exposed hESCs. Recently, miRNA were implicated in regulation of p21 translation in hESC [92]. It seems plausible to assume that the levels of p21 protein in irradiated hESCs are kept low to prevent interference with the cyclin-dependent kinases, such as CDK2, resulting in a non-operational G(1)/S checkpoint after IR. In turn, by not executing G(1)/S cell cycle arrest after IR, hESCs could evade spontaneous differentiation. To support this possibility, some recent data point to the G(1)/S phase as a primary timeframe when the hESCs fate decisions are made regarding the self-renewal and/or commitment to differentiate [29].

One of the key missions of DDR activation is to bring about DNA repair machinery to sites of DNA damage in order to preserve and maintain genomic integrity in all human cells. Both DDR and DNA repair share common and distinct molecular mechanisms and details of operation in hESCs and other types of human cells, such as fully differentiated ones.

### 4.2. DNA Damage response in Irradiated Human Mesenchymal Stem Cells

A network of signaling proteins representing sensors, mediators, transducers and effectors governs hMSC responses to IR. P53 was found to be activated as early as 1 min following high, 4 Gy dose of X-rays. Markedly, low doses of IR up to 0.1 Gy fail to induce p53, at least up to 30 min post-IR exposures.

High doses of IR were found to robustly induce p53 protein in hMSC cultures; after 20 Gy of gamma-radiation, p53 protein level remained elevated even after 6 days post-IR even though there was no significant changes in TP53 mRNA amount [51]. The activation of p53 measured by protein phosphorylation on ser 15 and ser 392 peaked at first day following IR, and then subsided by day six post-IR. Even relatively low doses of IR (0.5 Gy) elicited p53 induction in hMSC cultures [47]. Posttranslational modifications of p53 on ser 15, Chk1 on ser 345 and Chk2 on thr 68 were found to be strongly dependent on ATM function [46]. Expression of Hdm2 correlated well with that of p53; Hdm2 is activated by phosphorylation at serine 166 in hMSCs post-IR [51]. The number of γ-H2AX IRIF increased profoundly within an hour following IR and almost returned to levels found in mock-treated hMSCs within 24 h post IR. However, all hMSCs were bearing residual γ-H2AX IRIF even on day three after 20 Gy of IR indicating persistent DDR activation in such hMSC cultures which, in turn, may drive the execution of IR-induced senescence program [51].

### 4.3. DNA Damage Response in Irradiated Human Hematopoietic Stem Cells

The activation of DDR in hHSCs measured by levels of γ-H2AX IRIF showed a protracted resolution of DNA damage in these cells. Significantly more IRIF remained in hHSC subpopulations (average 7.1 per nucleus) compared to more mature progenitors (average 2.7) at 12 h post 3 Gy of IR [65]. Oxidative stress in these cells (other than IR-induced) was shown to robustly activate ATM, p53, 53BP1, CHK2 and FOXO3a resulting in an impairment of functional capabilities of oxidatively damaged hHSCs [93]. Importantly, accumulation of DNA damage was observed, and hHSCs were undergoing senescence-like arrest [93]. This is in a marked contrast to IR-induced apoptosis in hHSCs, described elsewhere [63,65,66]. Additional studies are warranted to be performed to reconcile these apparent discrepancies.

## 5. DNA Repair in Human Stem Cells after Exposures to Ionizing Radiation

### 5.1. DNA Repair in Irradiated Human Embryonic Stem Cells

The key DNA repair pathways known to function in human cells involve base excision repair (BER) [94,95], nucleotide excision repair (NER) [96,97], mismatch repair [98], non-homologous end-joining (NHEJ) [99,100], and homologous recombination repair (HRR) [101,102]. Multiple publications in the literature imply BER in correction of relatively small DNA lesions, such as oxidized bases, and/or alkylating agent damage. Interestingly, the efficacy of BER was recently demonstrated to depend upon the time of incubation of hESCs in culture being dependent on APE1 abundance [103]. During long-term culture, down-regulation of APE1 in hESCs is shown to result in partial failure of BER affecting the genomic stability in these cell cultures [103]. Another DNA repair activity, NER, is shown to remove mainly bulky alterations, such as those created following UV exposures. The findings on NER operating in hESCs available in the published literature are conflicting and inconsistent: the activity of NER in hESCs exposed to UV radiation was found to be modest at best, and as such insufficient to cope with the increased incidence of mutations in such stressed cells [104], whereas pluripotent cells were shown to possess high NER capacities [105]. Such discrepancies could be explained, at least in part, based on the different types of hESC lines used in these studies, equipped with different NER capabilities, various levels of genotoxic stress exposures, *etc.*

DNA double-stranded breaks (DSBs) are repaired with either HRR, or NHEJ [106]. HRR, which is essentially error-free, requires a homologous DNA template and as such operates in late S/G(2); in marked contrast, NHEJ is not dependent on a sequence homology to repair DSBs, which makes this pathway more error-prone. Mismatch repair is known to protect the genome against the accumulation of mismatched bases or single-stranded loops of DNA. The fidelity of DNA repair is of paramount importance, since unfaithful DNA repair could increase the chances for cell mutations and transformation to occur [107]. That’s why the information regarding the molecular mechanisms of DNA repair and details of their functioning in hESCs is so vital for future cell-based replacement therapies, as well as for basic knowledge on how pluripotent human stem cells such as hESCs manage to preserve their genomic stability in order not to pass the genetic errors to the subsequent generations.

Over the last few years, the accumulating evidence in general suggests that hESCs possess the overall increased efficacy of removing DNA damage compared to fully differentiated human cells [86,105,108–110]. DNA DSB repair at a targeted break site occurs with a much higher accuracy in hESCs than in somatic human cells [110]. Directed differentiation of hESCs into astrocytes decreased both the efficacy and fidelity of DNA repair. The frequency of an HRR event at a single DNA DSB differs up to 20-fold between otherwise isogenic hESCs based on the site of the DSB within the genome [110]. Hence, both the location of the site of damage within the genome and the stage of cell differentiation could determine the outcomes of DNA DSB repair.

DNA DSB repair in hESCs could be more complex than that found in both in neural progenitors (NPs) and astrocytes. The resolution of DNA DSBs examined with the gamma-H2AX focus assay was found to occur at a slower rate in hESCs compared to NPs and astrocytes [86]. However, both hESCs and NPs possess high capacity for HRR as judged by the dynamics of RAD51 foci. Surprisingly, ATM kinase was shown to be dispensable for IRIF formation in hESCs, whereas ATR was absolutely necessary for HRR activity [86]; the pattern reverted to the opposite upon hESCs differentiation. These data suggest that DDR may function and execute differently in hESCs and non-pluripotent human cells, even though the molecular components and pathways could be shared.

It was shown that both HRR and NHEJ are functional in hESC to perform DNA DSB repair, but HRR dominates. NHEJ kinetics is several-fold slower in hESCs and NPs than in astrocytes derived from hESCs [109]. Intriguingly, NHEJ in hESCs is largely independent of ATM, and PARP; it appears, however, to be dependent on XRCC4 with repair fidelity several-fold greater in hESCs than in astrocytes [109]. The degree of involvement of DNA-PKcs in NHEJ in hESCs is contradictory: on the one hand, DNA-PKcs was found to be responsible for elimination of IR-induced chromatid breaks in late G(2) phase of cell cycle [111]; on the other hand, DNA-PKcs knock-down in hESCs was not associated with any major alterations in NHEJ [109]. Therefore, the role of DNA-PKcs in DNA repair in hESCs still needs to be clarified.

Compared with human primary fibroblasts, different types of DNA damage induced by IR exposures, H_2_O_2_, UV-C, and psoralen are repaired more efficiently in hESCs cultures [108]. DNA microarray gene expression analysis revealed that transcript levels of several DNA repair genes are increased in hESCs compared with their more differentiated descendants found in embryoid bodies [108].

Recently, it was shown that DNA repair capacities of hESCs and induced pluripotent (iPSCs) cell lines are more heterogeneous than those of differentiated cell lines [105]. Notably, even low levels of UV exposures induced an apoptotic response in hESCs, although small subpopulations survived, accumulating point mutations with a typical UV signature, and possessing more resistance to induction of apoptosis following subsequent genotoxic stress exposures [104]. Interestingly, human pluripotent cells that survived UV exposures exhibited less DNA damage compared with differentiated cells that received the same UV flux [105].

The majority of data indicate that the mechanisms underlying the genomic stability are in general enhanced in hESCs, relative to differentiated human cells. Still, examinations of the DNA repair capacities in given hESC lines, and characterization of their genomic stability prior to any possible application in clinical trials in future remains a top priority for regenerative medicine.

### 5.2. DNA Repair in Irradiated Human Mesenchymal Stem Cells

DNA DSBs were found to be repaired within 12–16 h post 5 Gy of IR exposures in hMSCs [48]. It was more rapid in undifferentiated hMSCs than in differentiated osteoblasts judged by using γ-H2AX IRIF foci as a surrogate marker for DNA DSBs after IR. The levels of nuclear Ku70 increased substantially and peaked 2 h post 5 Gy of IR; activation of ATM and DNA-PKcs was maximal between 30 min and 2 h under these IR conditions [48]. These data suggest the involvement of NHEJ repair mechanism in IR-exposed hMSC cultures. NER was also shown to be operational in hMSCs [61]. Importantly, the sequential transformation of hMSCs is associated with the reduction in DNA DSB repair capacity and increased radiosensitivity, which can at least partly be connected to enhanced DNA repair checkpoint signaling in such hMSCs [112].

### 5.3. DNA Repair in Irradiated Human Hematopoietic Stem Cells

DNA DSB repair was significantly delayed in hHSCs irradiated with high doses of exposure. Strikingly, only less than 1% of DSB were rejoined in quiescent hHSCs within the first 30 min post 15 Gy of IR; at 12 h post 3 Gy, 82% of hHSCs were still bearing increased numbers of IRIF indicative of persistent DNA DSBs in these cells [65]. Interestingly, in murine HSCs the state of quiescence was recently shown to predispose these cells to error-prone NHEJ and mutagenesis [113]; whether the same is true for hHSCs remains to be determined. The presence of thrombopoietin was shown to be critical for DNA repair to occur in hHSC; this finding underscores the importance of microenvironmental factors in regulating the physiological responses of hHSCs to genotoxic stressors such as IR exposures [114].

### 5.4. DNA Repair in Irradiated Human Neural Stem/Progenitor Cells

IRIF assay for gamma-H2AX showed that DNA DSB repair is functional in hNSC cultures. The well-discernible gamma-H2AX foci were observed in hNSC when analyzed 20–30 min after irradiation; by 3 h after initial IR exposure, IRIF levels reached those seen in non-IR treated cells [71]. There is an indication that human neural progenitors may rely on homologous recombination DNA repair (HRR); at 12 h post-2 Gy of IR exposures, 65% of cells contained Rad51 IRIF indicative of HRR [86]. In human neural progenitors, NHEJ was shown to be of a higher fidelity (up to 1.8-fold) compared to more differentiated brain cells such as astrocytes [109].

## 6. Transcriptional Responses of Cultured Human Stem Cells to Ionizing Radiation

### 6.1. Changes in Gene Expression in Irradiated Human Embryonic Stem Cells

The transcriptional responses of many differentiated somatic human cells exposed to IR have been extensively characterized in the past [115–117]. However, this is not true regarding the signaling networks underlying global regulation of transcriptional outputs in pluripotent hESCs. Only recently have emerging reports in the literature begun to fill gaps in such knowledge

One of the most important findings from gene expression profiling in irradiated hESCs is that the expression set of core transcription factors underpinning pluripotency in hESCs is not changed significantly by IR exposures at any dose up to 4 Gy of gamma-radiation [18]. IR-exposed hESCs initiate induction of gene sets broadly involved in p53 signaling, cell death, cell cycle, embryonic and organ development, and others.

Modest dose (0.4 Gy) of gamma-radiation was found not to elicit the overexpression of *CDKN1A* and *HDM2* which is in marked contrast with gene expression programs triggered by IR in fully differentiated human cells [118]. Irradiation of hESCs with 2 Gy dose results in changes in expression of genes belonging to canonical Wnt/β-catenin and TFG-β signaling, which may potentially lead to major perturbations in hESCs cultures. Notably, 2 Gy dose of radiation induces *CDKN1A* overexpression by 2.3-fold, but fails to trigger the upregulation of some other p53-regulated genes, such as *HDM2*. The general metabolism pathways’ genes such as amino acid metabolism, molecular transport, and cell morphology, were found to be modulated in hESCs cultures by 2 Gy of IR. Compared to 2 Gy dose, the general trend in gene expression alterations was about the same after 4 Gy of IR exposures, but a distinct subset of genes related to organ and tissue development was identified to have a changed expression. After 4 Gy irradiation, p53 and aryl hydrocarbon signaling pathways, and functions involved in cell death, cell cycle, proliferation, and embryonic development were found to be statistically significantly affected in hESCs cultures. *TP53INP1*), *CDKN1A* and *HDM2* were identified to be responsive after IR exposures with 4 Gy dose. A few minor alterations occurring in the development and differentiation processes with 4 Gy of irradiation in hESCs appear to be insufficient to cause loss of pluripotency since the successful formation of teratomas from 4 Gy-irradiated hESCs was readily observed. Importantly, the transcriptional changes in hESCs are found to be dose-dependent at 24 h after IR.

At about the same time, our group demonstrated that the changes in gene expression in hESCs after IR exposures are principally different from those observed in somatic human cell lines [87]. Early after IR, the gene expression signature featured almost an exclusively p53-dependent, clearly pro-apoptotic, transcriptional response with a total of only 30 up-regulated genes, such as *BTG2*, *CDKN1A*, *SESN1*, *IER5*, and *GADD45A*, that are known to be radioresponsive in human somatic differentiated cells [116,117,119]. The induction of these genes is often considered to be associated with temporal cell cycle arrest (*GADD45A, PLK2* and *PLK3* are all involved in G(2)/M checkpoint). Among other genes robustly overexpressed 2 h post IR are some pro-apoptotic genes, such as *GDF15*, *BBC3*, *HTATIP2*, *CARD8*, *FAS*, *TP53INP1* and transcription factors belonging to zinc finger protein superfamily (*ZNF79*, *ZNF761*, *ZSCAN20*, and *ZNF135)*. Importantly, the analysis of transcription patterns at 16 h post IR revealed 354 differentially expressed genes in hESCs, with many genes involved in pro-survival pathways, such as metallothioneins (*MT1M*, *MT1L*, *MT1H*, and *MT1G*) [116,117,119], and general metabolism signaling. A few members of the histone gene superfamily, such as *HIST1H4I* and *HIST1H4E*, were found to be strongly IR-responsive at 16 h. Among other genes upregulated at 16 h post IR were many transcription factors (*ZNF302*, *SP5*, *ZNF33A*, *ZNF697*, and *ZFYVE16*). All of the late response genes were found to be overexpressed, with the magnitude of expression being in a range between 1.5-fold and 25-fold over sham-treated cell cultures.

In summary, the gene expression signatures characterizing early (2 h) and late (16 h) radioresponse of hESCs cultures to 1 Gy of IR are distinct [87]. Of note, only six genes (*CDKN1A*, *BTG2*, *GDF15*, *SESN1*, *PLK3* and *ANKRA2*) were found to be differentially expressed at both timepoints examined. Whether such a “gene expression signature” could be used as a biomarker of IR exposure of hESCs needs to be addressed in further studies with more comprehensive dose-response, time-course and hESCs line-specific analyses. Such biomarkers may potentially include not only defined transcriptional signatures, but also refined patterns of DNA/histone chemical modifications and non-coding RNAs constituting together the epigenetic profile of IR-exposed hESCs.

### 6.2. Gene Expression Alterations in Irradiated Human Mesenchymal Stem Cells

The transcriptional responses of hMSC induced by low and modest doses of ionizing radiation examining the dynamics of gene expression changes were recently illuminated in several studies. The gene expression changes were in general modest after 1 Gy of IR in hMSCs, especially during the early response at 5 h post-IR (less than 10 genes in total) [44]. One of the major changes observed was the downregulation of cyclin E2 (CCNE2), concomitant with the ongoing cell cycle arrest at this timepoint. The late response was more robust (up to 174 genes modulated), but there was no clear dose-response relationship. For example, low X-ray dose in 0.1 Gy elicited the alterations in 144 genes, and higher dose 1 Gy modulated only 129 genes [44]. Several pathways, including IGF-1 signaling, integrin signaling, cytoskeleton signaling, estrogen receptor signaling, and insulin receptor signaling, were enriched specifically for low-dose (0.1 Gy) X-rays. In marked contrast, cell cycle regulation and DNA/RNA metabolism were overrepresented for both high-dose (1 Gy) X-rays and ^56^Fe ions [44]. One of the most prominent markers of IR exposures, *CDKN1A*, was shown to be upregulated by 2.1-fold by 1 Gy ^56^Fe ions compared with 1.8-fold by 1 Gy X-rays, relative to mock-treated controls.

The effects of low doses of IR in transcriptional responses in hMSCs were examined in [120]. Immortalized hMSC were exposed to IR with 0.01, 0.05, 0.2 and 1 Gy of gamma radiation and transcriptomic analysis was carried out with total RNA extracted from each hMSC line at 1, 4, 12 and 48 h following IR exposures. It was found that a total of 6016 genes were differentially expressed at more than one time point or dose level. Genes with dose-dependent transcriptional alterations were involved in signal transduction, proteolysis, peptidolysis, regulation of transcription, and metabolism. Importantly, analysis of dose-dependent group of genes revealed a highly non-linear relationship between the IR dose and the transcriptional output. The time-dependent set of transcripts also exhibited a non-linear relationship. Some of the early-response genes (up to 4 h post-IR) showed a differential expression to 0.01, 0.05 and 0.2 Gy but were unresponsive to relatively high 1 Gy dose. Some of the late-response (12–48 h) genes showed a differential expression to 1 Gy but were relatively unresponsive to other doses [120]. These observations underscore the complexity of transcriptional responses of hMSCs to IR, and prompt further studies to clarify the key signaling pathways governing such responses.

## 7. The Role of Epigenetics in Responses of Human Stem Cells to Ionizing Radiation Exposures

Some changes in gene expression and/or alterations in phenotype caused by mechanisms other than alterations in the nucleotide sequences of the genomic DNA are known to be heritable being a result of DNA methylations and/or histone posttranslational modifications. Very limited information is available on the role of epigenetics in radioresponses of hESCs. Importantly, the unique features of chromatin “openness” and relative lack of heterochromatin in human pluripotent cells suggest that the findings on epigenetics involvement in radioresponse obtained with human somatic differentiated cells [121–123] could not be easily extrapolated to hESCs. Notably, the association of the histone bivalent marks with promoters of many developmentally regulated genes, and a much larger abundance of non-CG DNA methylation observed in hESCs [124–127] makes a difference which is likely to affect the radioresponses in pluripotent stem cells.

The changes in the global microRNAome in IR-exposed hESCs constitute yet another level of epigenetic regulation. MicroRNAs (miRNA) were previously implicated in regulation of key biological processes and functions at the post-transcriptional level. It was found that many miRNA species are expressed predominantly in hESCs [128,129] and genotoxic stresses, such as UV-exposures, result in differential expression of many miRNA species (e.g., *miR-302* cluster, *miR-371-372* family genes) [130].

Recently, we showed for the first time, that the miRNAome undergoes global alterations in hESCs after IR. The comprehensive interrogation of expression levels of 1090 miRNA species in hESCs showed statistically significant changes in 54 genes following 1 Gy of X-ray exposures [131]; the global miRNAome alterations are highly temporally and cell line-dependent in hESCs. The magnitude and the level of induction of IR-responsive miRNA species in hESCs is much more robust at 16 h post-IR of hESCs compared to “early” 2 h. Gene Ontology analysis reveals that miRNAome changes post-IR aim to maintain the pluripotent state of surviving irradiated hESCs; most notably, these are associated with the cell cycle-, and alternative splicing-related processes.

## 8. The Non-Targeted Effects of Ionizing Radiation on Human Stem Cells

The non-targeted effects of IR exposures include adaptive responses, low-dose hypersensitivity, genomic instability, and so-called bystander effect (RIBE). RIBE was studied in a number of experimental systems both *in vitro* and *in vivo* in the past; it manifests itself by intercellular communication from irradiated cells to non-irradiated cells which may cause a plethora of biological effects mimicking those observed in directly hit cells, in these bystander cells. To date, very little is known about RIBE and other non-targeted radiation effects in hSC. To close this gap in knowledge, we recently examined RIBE in both hMSCs and hESCs irradiated with doses 0.2, 2 and 10 Gy of X-rays [50]. Our data showed no evidence for RIBE either in hMSC and hESC by the criteria of induction of DNA damage and for apoptotic cell death compared to non-irradiated cells. These findings indicate that hSC might not be susceptible to damaging effects of RIBE signaling compared to differentiated adult human somatic cells as reported previously.

## 9. The Effects of Ionizing Radiation on Human Colon/Intestinal Stem Cells

The nature and origin of human gastrointestinal (GI) tract stem cells (ISCs) are still under investigation (for review, see [15]). This partly explains why the data on biological effects of IR on human colon/ISCs are very limited; most of research in this area was focused on rodent studies. The epithelium lining the small intestine undergoes rapid regeneration supported by crypt intestinal stem cells (ISCs). Recently, it was found that Bmi1+ and Lgr5+ are two functionally distinct long-lived multipotent ISCs in mice. Myb- and Wnt-regulated Lgr5+ marks continuously cycling ISCs that contribute to homeostatic regeneration [132], and are radiosensitive [133,134]; this effect was not seen in the duodenum [134]. Lgr5+ is a cell surface receptor protein with the corresponding ligand remaining elusive; its key role is thought to be delimiting stem cell pool within the respective niche [135]. Interestingly, very recently Lgr5+ cells were shown to be very efficient in reconstituting the mammary glands, therefore not being the stem cell marker unique for ISCs [136]. The gene expression signature of Lgr5+ reveals one of the key Wnt targets, namely the Achaete Scute-like 2 (Ascl2) transcription factor controlling intestinal stem cell fate; deletion of Ascl2 was shown to result in exhaustion of the Lgr5+ stem cell compartment within days [137]. Recently, it was suggested that Ascl2 could exert its effect through *miRNA-302b* related pathways [138]. In marked contrast, Bmi1+ quiescent ISCs are quite resistant to high-dose IR. Importantly, after IR the normally quiescent Bmi1+ ISCs proliferate extensively to clonally repopulate crypts and villi [133]; this could involve activation of cyclinE1 [132]. Therefore, IR-triggered damage may not accumulate in the colonic Lgr5^+^ stem cells *per se*. Crypt base columnar stem cells (CBCs) residing in the small intestine in mice were also shown to be relatively radioresistant, undergoing apoptosis less than 24 h after high doses of IR exposures or experiencing mitotic cell death thereafter [139]. The molecular pathways responsible for colon/intestinal SC killing could involve the induction of p53 and proapoptotic PUMA which could be suppressed by overexpression of Akt and growth factors such as bFGF and IGF-1 [140]. CBCs effectively operate DNA HRR protecting them from IR toxicity. Survival of CBCs at 2 days predicts crypt regeneration in the following days, and ensures whole body survival from gastrointestinal syndrome [139]. The totality of available data illuminates the crucial role of multipotent colon/ISCs in mitigating IR-induce damage in GI.

## 10. Conclusions

The biological responses of hSCs to IR have become an object of intense research over the last decade, as overviewed here. Human adult stem cells are characterized by very heterogeneous responses to IR; exemplified from radioresistant hMSCs to highly radiosensitive hHSCs. Importantly, even though many molecular details on responses of hSCs to IR exposures are already uncovered, there is still lack of information on how the defining features of adult hSC, namely self-renewal and multipotency, are affected by IR. The situation is complicated by a large body of controversies that exist in published reports regarding response of hSCs to IR; various sources of stem cells, incomplete characterization of starting isolates of cell cultures, inherent heterogeneity of hSC populations, lack of integrated studies involving modern “omics” approaches, and other factors compound the problem. But based on cell culture research, it appears that the radiation syndromes involving human cell toxicity depending on a dose of exposures could at least partly be governed by the exhaustion/dysfunction of corresponding irradiated hSC compartments, and dysregulated hSC homeostatic mechanisms. On the other hand, hESCs possess a unique, distinct from adult hSCs, radioresponse, perhaps associated with hESCs exquisite characteristics as pluripotent human cells. Human ESCs rapidly undergo apoptotic cell death following even relatively modest IR exposures (0.2 Gy and higher), but a surviving fraction of these cells could still give rise to derivatives of all three germ layers, and importantly, could potentially carry the increased load of mutations, as shown in the case of UV radiation damage [104]. The key question remains as to whether the damage inflicted by low dose/low dose rate-protracted IR exposures would result in developmental/genetic and or epigenetic abnormalities as a delayed response to IR. If translated into an *in vivo* situation, the ramifications of such research would be broad covering many aspects of human health, risk assessment and possibly even environmental protection guidelines.
